# Plasminogen activator inhibitor‐1 serum levels in frontotemporal lobar degeneration

**DOI:** 10.1111/jcmm.18013

**Published:** 2024-02-22

**Authors:** Francesco Angelucci, Katerina Veverova, Alžbeta Katonová, Martin Vyhnalek, Jakub Hort

**Affiliations:** ^1^ Memory Clinic Department of Neurology Second Faculty of Medicine Charles University and Motol University Hospital Prague Czech Republic; ^2^ International Clinical Research Centre St. Anne's University Hospital Brno Czech Republic

**Keywords:** dementia, frontotemporal lobar degeneration, plasminogen activator inhibitor‐1, tissue‐type plasminogen activator

## Abstract

Plasminogen activator inhibitor‐1 (PAI‐1) impedes brain plasmin synthesis. Reduced plasmin activity facilitates cumulation of amyloid beta (Aβ) in Alzheimer's disease (AD). Since plasmin also regulates the synaptic activity, it is possible that altered PAI‐1 is present in other neurodegenerative disorders. We investigated whether PAI‐1 and its counter‐regulatory tissue plasminogen activator (tPA) are altered in serum of patients with dementia due to frontotemporal lobar degeneration (FTLD). Thirty five FTLD patients (21 in mild cognitive impairment stage (MCI) and 14 in dementia stage) and 10 cognitively healthy controls were recruited. Serum tPA and PAI‐1 protein levels were measured by anova. Correlation between biochemical and demographic data were explored by measuring Pearson correlation coefficient. Serum PAI‐1 levels were elevated in the FTLD dementia group as compared to FTLD MCI and controls. tPA serum levels and PAI‐1/tPA ratio did not significantly differ among groups. There was a negative correlation between PAI‐1 serum levels and disease severity measured by MMSE score. No correlations of tPA serum levels and PAI‐1/tPA ratio with MMSE were found. Increased PAI‐1 serum levels may serve as a marker of dementia in FTLD, suggesting that, besides Aβ pathway, the plasmin system may affect cognition through synaptic activity.

## 1 INTRODUCTION

Plasmin is the leading enzyme in the fibrinolytic system, a biological process responsible for the dissolution of fibrin clots formed because of vascular lesions.^1^ However, in recent years, new and unexpected functional roles for this system have been identified mostly in relation to the central nervous system (CNS) that are unrelated and independent of fibrin degradation and clot removal.^2^, ^3^

Plasmin synthesis in the brain is regulated by two enzymes^4^: one activating, the tissue plasminogen activator (tPA), and the other inhibiting, the plasminogen activator inhibitor‐1 (PAI‐1).^5^ These enzymes can be produced by various elements of the CNS, including neurons, astrocytes and oligodendrocytes.^4^, ^6^, ^7^ Indeed, plasminogen/plasmin system and its modulators tPA and PAI‐1 have been shown to not only have critical roles in modulating astrocytes, neurons, microglia and pericytes but also to have profound effects in several CNS conditions, including ischaemic stroke,^2^ severe traumatic brain injury^8^ and also in neurodegenerative disorders such as Parkinson's^9^ and Alzheimer's^10^ diseases.

As for neurodegenerative diseases, previous studies indicate that in Alzheimer's disease (AD) there is an imbalance of tPA and PAI‐1 that leads to reduced plasmin synthesis and activity. Low plasmin levels were observed in 67% of AD *APOE4* brains.^11^ Furthermore, in two transgenic AD mouse models (Tg2576 and TgCRND8), it was observed that accumulation of amyloid beta (Aβ) peptide in the brain was associated with the upregulation of PAI‐1 and inhibition of tPA.^12^ In another brain tissue post‐mortem study, it was shown that both PAI‐1 and tPA were elevated in AD patients, suggesting that there is not alteration of the synthesis of plasmin.^13^ However, it was suggested that the increase in PAI‐1 could have consequences in neuronal activity, including synaptic deregulation and excitotoxicity. In another study, plasmin and its precursor plasminogen were not altered in post‐mortem AD versus the control brain tissue.^10^ However, reduced plasmin activity was observed in AD brains,^10^ indirectly favouring the accumulation of Aβ in aggregate or soluble forms.^11^

In a previous recent study, we have shown that while tPA serum levels were unaffected, PAI‐1 levels increased in patients with dementia due to AD and its prodromal stage amnestic mild cognitive impairment (aMCI).^14^ In addition, there was a negative correlation between PAI‐1 serum levels and Mini‐Mental State Examination (MMSE) score. In fact, PAI‐1 levels were higher in patients with dementia due to AD than in aMCI patients and even more than in cognitively healthy controls.^14^ Similarly, but to a lesser extent, the ratio between PAI‐1 and tPA gradually increased from controls to patients with aMCI and those with AD dementia.^14^

The role of plasmin in AD appears to be related to the ability to reduce the Aβ in its soluble forms.^15^, ^16^ These data suggest that in AD dementia, an imbalance of these two enzymes may favour the accumulation of Aβ and therefore AD.^10^, ^11^ Despite this, we do not know if defects in the plasminogen/plasmin system are also associated with dementia in other non‐AD pathologies and, therefore, unrelated to the accumulation of Aβ in neurons.

Frontotemporal dementia (FTD) represents a group of clinically defined brain disorders characterized by prominent behavioural/cognitive symptoms like disinhibition, apathy, executive deficits and language disorders. Several clinical forms have been recognized based on the localisation of neurodegeneration and subsequent clinical manifestation.^17^ FTD is associated with several underlying neurodegenerative diseases characterized by frontotemporal lobar degeneration (FTLD).^18^ FTLD is characterized by deposits of tau or other proteins but, contrary to AD, intracerebral deposition of Aβ is not a typical hallmark of FTLD.^19^, ^20^ Thus, subjects affected by FTLD represent a group of patients with dementia unrelated to the Aβ pathophysiological accumulation present in AD.

Thus, in this study we investigated these enzymes regulating plasmin synthesis in FTLD patients in mild cognitive impairment (FTLD‐MCI) and dementia (FTLD dementia) and in cognitively healthy controls. Our aim was to establish whether serum levels of tPA and PAI‐1 were altered in FTLD patients and whether there is a correlation with MMSE score.

## 2 MATERIALS AND METHODS

### 2.1 Participants

Thirty one FLTD subjects from the Czech Brain Aging Study, a longitudinal, memory clinic‐based study on aging and cognitive impairment,^21^ and 10 cognitively healthy participants were included in the study.

Among these subjects, 14 FLTD subjects were in the dementia stage (FTLD dementia–six patients with a semantic variant of primary progressive aphasia, five patients with a behavioural variant of FTD, two patients with FTD‐amyotrophic lateral sclerosis and one patient with a non‐fluent variant of primary progressive aphasia).

The remaining 21 patients were in MCI stage (FTLD MCI–5 patients with a semantic variant of primary progressive aphasia, 12 patients with the behavioural variant of FTD, 1 patient with FTD‐amyotrophic lateral sclerosis, and 5 patients with non‐fluent variant of primary progressive aphasia). The main criterion to diagnose the dementia stage was based on the impairment of activities of daily living reported by the patient's informant during the structured interview. Diagnoses of bvFTD was based on Rascovski criteria^22^ and language variants were diagnosed based on Gorno‐Tempini criteria.^23^ The diagnosis of the MCI stage was based on Petersen's criteria for MCI, including cognitive complaints, evidence of memory dysfunction on neuropsychological testing, generally intact activities of daily living, and absence of dementia.^24^ All participants underwent standard neurological and laboratory evaluations, comprehensive neuropsychological examination, and 1.5‐T brain magnetic resonance imaging (MRI) within 3 months from the initial visit. The majority of participants underwent cerebrospinal fluid (CSF) sampling with analysis of AD biomarkers (*n* = 40). FTLD patients (*n* = 33) had CSF negative for amyloid biomarkers (normal Aβ42), and healthy controls (*n* = 7) were negative for all three AD biomarkers (Aβ42, T‐tau, P‐tau181) based on internal cut‐offs. **Individuals who did not donate CSF (*n* = 5) underwent A**β **PET imaging with a negative A**β **load.** The results of biomarker analyses are summarized in Table 1. All participants in this study signed written informed consent approved by the Motol University Hospital ethics committee.

**TABLE 1:** Clinical and demographic characteristics of FTLD patients and cognitively healthy controls.

Parameter	FTLD dementia patients (*n* = 14)	FTLD MCI patients (*n* = 21)	Healthy controls (*n* = 10)	Statistics
Age (years)	64.4 ± 8.3	66.7 ± 5.0	61.2 ± 12.2	*p* = 0.498
Sex (male/female)	9 M/5 F	14 M/7 F	3 M/7 F	*chi‐square* *p* = 0.129
BMI	25.8 ± 4.6	25.4 ± 2.1	25.5 ± 4.7	*p* = 0.956
Years of education	14.7 ± 2.6	15.3 ± 3.3	18.1 ± 3.8	*p* = 0.190
MMSE	20.9 ± 6.3**	24.9 ± 3.8*	29.9 ± 0.3	*p < 0.05 vs. Controls **p < 0.001vs. Controls
Aβ 42/40 ratio	0.08 ± 0.02	0.09 ± 0.02	0.08 ± 0.02	*p* = .506
Aβ 42 in CSF, pg/ml	758.4 ± 320.4	783.8 ± 332.7	881.3 ± 228.0	*p* = .693
T‐tau in CSF, pg/ml	414.0 ± 218.4	314.1 ± 143.2	274.7 ± 110.2	*p* = .151
P‐tau 181 in CSF, pg/ml	53.3 ± 38.5	36.3 ± 14.5	35.0 ± 13.2	*p* = .134
NfL in serum, pg/ml	72.2 ± 56.8	57.0 ± 63.0	16.0 ± 5.3	*p* = .139
Ng in CSF, pg/ml	202.6 ± 70.1	162.2 ± 74	182.8 ± 55.6	*p* = .355

*Note*: Data are the mean ± standard deviation. N = number of subjects included in the study.

* indicates a significant difference between the groups. *p < 0.05; **p < 0.001.

Abbreviations: Aβ, amyloid beta; BMI, body mass index; F, female; FTLD, frontotemporal lobar degeneration; M, male; MCI, mild cognitive impairment; MMSE, Mini Mental State Examination; NfL, neurofilament light chain; Ng, neurograninP‐tau 181, tau phosphorylated at threonine 181; T‐tau, total tau.

### 2.2 Exclusion criteria

The participants were not included in the study if they had a history of neurological or psychiatric disorders other than FTLD potentially causing cognitive deficit (i.e. history of stroke, Parkinson's or Alzheimer's disease, brain tumour, alcohol abuse), hearing difficulties, depressive symptomatology (≥6 points on the 15‐item Geriatric Depression Scale)^25^ or had significant vascular impairment on the brain MRI (Fazekas scale more than 2).^26^

### 2.3 Blood and CSF sampling

Blood samples were drawn by venipuncture. After collection of the whole blood, the blood was allowed to clot by leaving it undisturbed at room temperature for 15 min. Blood samples were then centrifuged at 1700 × ***g*** at 20°C for 5 min. All samples were centrifuged within 30 min of collection. Serum supernatant was collected, divided into 0.5 mL polypropylene aliquot tubes, and kept frozen at −80°C until further use.

CSF was obtained by lumbar puncture in a supine position. The pattern of CSF collection was the same for all study individuals, with the patient lying on their side, and CSF drawn at the L3/L4 or L4/L5 region and collected in 8‐ml polypropylene tubes. Each sample was then combined, gently mixed, and centrifuged within 30 min of lumbar puncture at 1700 × ***g*** at 20°C for 5 min. CSF was aliquoted in polypropylene tubes of 0.5 mL and stored at −80°C until analysis.

Before biomarker analysis, serum and CSF samples stored at −80°C were thawed and vortexed for 15 s.

### 2.4 Immunological assays

AD biomarkers in CSF (Aβ42, T‐tau, and P‐tau181) were analysed using ELISA (Innogenetics, Ghent, Belgium) in the Cerebrospinal Fluid Laboratory, Institute of Immunology and Department of Neurology, Second Faculty of Medicine, Charles University and Motol University Hospital. Unbiased cut‐offs of less than 665 pg/mL and more than 48 pg/mL and 358 pg/mL were used to define amyloid‐b1–42, p‐tau181, and total tau positivity, respectively. These predefined cut‐offs^27^, ^28^ were based on internal receiver operating characteristic (ROC) analyses and were validated against amyloid PET status in the Czech Brain Aging Study with 79% agreement and areas under the ROC curves (AUCs) of 85%.^29^

Serum levels of PAI‐1 (Catalogue Number: DY1786) and tPA (Catalogue Number: DY7449) were measured with commercial ELISA kits from R and D Systems as previously described.^14^ All samples were tested in duplicate. Values were ng/mL for PAI‐1 and pg/mL for tPA.

### 2.5 PAI‐1/tPA ratio determination

Values of serum PAI‐1 were converted in pg/mL and used to calculate the PAI‐1/tPA ratio according to the following formula: PAI‐1 (pg/mL): tPA (pg/mL) = PAI‐1/tPA ratio.^30^

### 2.6 Statistical analysis

Serum levels of PAI‐1 and tPA and PAI‐1/tPA ratio among the groups were compared by anova and Fisher‐protected least post hoc. Demographic data were analysed by *Chi‐squared* test. Correlation between biochemical and demographic data were explored by measuring Pearson correlation coefficient. *p* <0.05 was considered statistically significant. The level of statistical significance was set at *p* < 0.05. Statistical analysis was performed by Statview (SAS Institute).

## 3 RESULTS

### 3.1 Demographic data

Table 1 shows demographic and clinical data. Groups did not differ in age (*p* = 0.169) and sex distribution (*chi‐squared p value* = 0.129).

The FTLD dementia and FTLD MCI patients had a lower level of education as compared to the healthy subjects (*p* < 0.05). No difference in educational level was observed between FTLD dementia and FTLD MCI (*p* = 0.592). FTLD dementia (*p* < 0.001) and FTLD MCI (*p* < 0.01) groups had lower MMSE score than controls. The FTLD dementia group had also lower MMSE score in comparison with FTLD MCI group (*p* < 0.05).

### 3.2 Serum levels of PAI‐1, tPA and PAI‐1/tPA ratio in FTLD dementia and FTLD MCI groups

**FIGURE 1:**
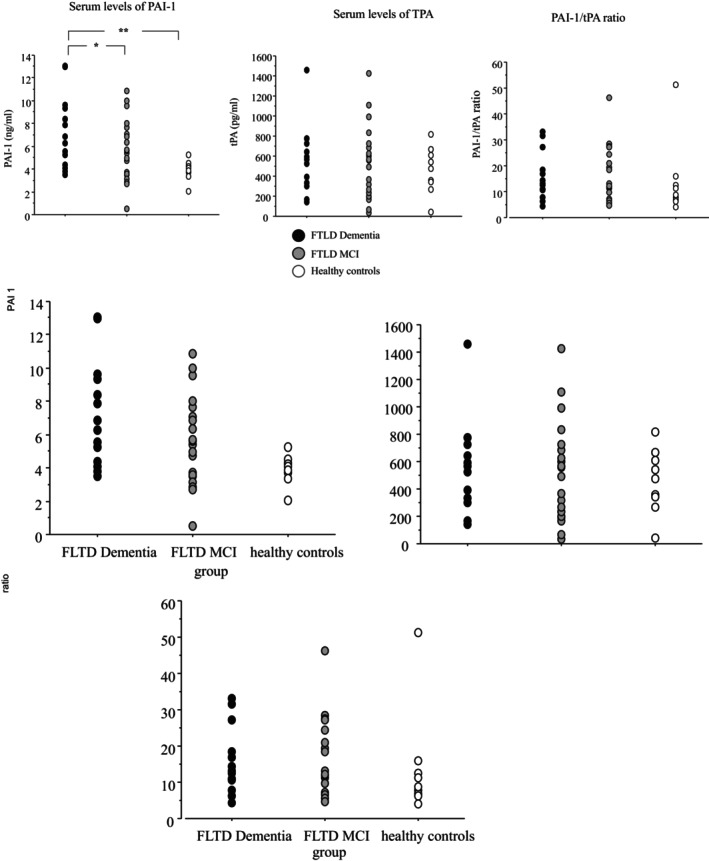
PAI‐1 and tPA serum levels, PAI‐1/tPA ratio in FTLD dementia and FTLD MCI patients and cognitively healthy controls. Data are the mean ± SEM. Values are expressed in ng/ml (PAI‐1) and pg/ml (tPA). Asterisk (*) indicates a significant difference between the groups. * *p* < 0.05; ** *p* < 0.01.

Figure 1 shows serum levels of PAI‐1, tPA and PAI‐1/tPA ratio in FTLD dementia, FTLD MCI and healthy subjects. anova showed a significant group effect in PAI‐1 levels (*p* < 0.01). The post hoc test revealed that PAI‐1 serum levels were significantly elevated in the FTLD dementia versus FTLD MCI (*p* < 0.05) and healthy subjects (*p* < 0.01). (Figure 1). The difference between FTLD MCI and controls was not significant.

There were no statistical differences in serum levels of tPA among the groups (*p* = 0.709). In addition, PAI‐1/tPA ratio was not significantly different among groups (*p* = 0.745) (Figure 1).

### 3.3 Correlations between PAI‐1/tPA serum levels, PAI‐1/tPA ratio and MMSE score

**FIGURE 2:**
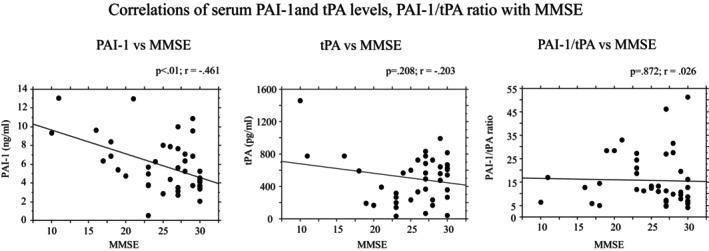
Correlations between serum levels of PAI‐1, tPA, PAI‐1/tPA ratio and disease severity (MMSE). *r* = Pearson correlation coefficient; *p* = p value.

As shown in Figure 2, there was a negative correlation between serum levels of PAI‐1 and MMSE score in the whole cohort of subjects included (*r* = −0.461, *p* < 0.01). No statistically significant correlations between tPA serum levels and MMSE (*r* = −0.203, *p* = 0.208) and between PAI‐1/tPA ratio and MMSE were observed (*r* = 0.026, *p* = 0.872) (Figure 2).

### 3.4 Correlations between PAI‐1/tPA serum levels, PAI‐1/tPA ratio and education level (years)

**FIGURE 3:**
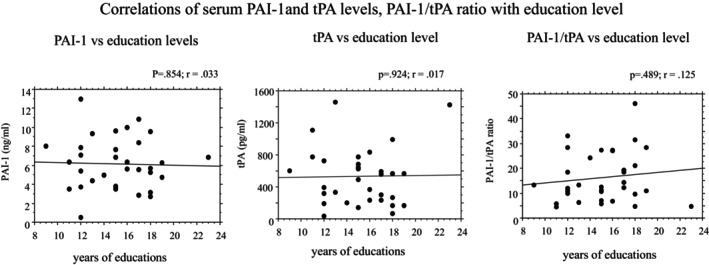
Correlations between serum levels of PAI‐1, tPA, PAI‐1/tPA ratio and education level. *r* = Pearson correlation coefficient; *p* = p value.

Correlations between PAI‐1/tPA serum levels, PAI‐1/tPA ratio and education level are shown in Figure 3. No statistically significant correlations of education levels with PAI‐1 (*r* = 0.033, *p* = 0.854), tPA serum levels (*r* = 0.017, *p* = 0.924) and PAI‐1/tPA ratio (*r* = 0.125, *p* = 0.489) were observed.

## 4 DISCUSSION

This study investigated whether patients with dementia due to FTLD have altered serum levels of tPA and PAI‐1 compared to those with MCI due to FTLD and controls. We found that FLTD dementia patients were characterized by increased levels of the inhibitor of plasmin PAI‐1 versus FTLD MCI and controls. In addition, PAI‐1 levels negatively correlated with the MMSE score.

These data align with our previous study showing that PAI‐1 levels in serum are increased in patients with AD dementia compared to aMCI and healthy controls.^14^ The present findings support the idea that the syndrome of dementia is associated with high PAI‐1 levels in serum, independently from the pathology causing dementia, which was AD in the mentioned study and FLTD in the present study.

High levels of PAI‐1 could mean that plasmin synthesis is reduced in dementia. In line with these data, it has been demonstrated that plasmin activity is decreased in AD animal models and humans.^10^, ^12^ Furthermore, in animal models of AD^31^ and cell cultures,^10^, ^32^, ^33^ when PAI‐1 is pharmacologically inhibited, plasmin increases its degrading activity on Aβ.

The present findings also suggest that increased PAI‐1 serum levels characterize even dementia not associated with Aβ accumulation. This assumption implies that, besides Aβ degradation, the plasmin system may be involved in cognitive functions through other signalling pathways in the CNS. In line with this hypothesis, it has been shown that the plasmin system is important in processes regulating cognitive functions in the CNS, such as synaptic plasticity,^4^ NMDA receptor‐mediated signalling^34^ and long‐term potentiation.^34^, ^35^ In this regard, it is worth mentioning that even the synthesis of essential proteins regulating synaptic activity, such as BDNF,^36^ may be regulated by plasmin activity in the CNS.^37^, ^38^, ^39^ It has been repetitively demonstrated that BDNF levels in the CNS and the periphery are altered during pathological conditions characterized by cognitive dysfunctions and dementia.^40^, ^41^ It would be interesting to verify whether circulating and brain levels of BDNF and PAI‐1 show some correlation. Besides this mechanistic hypothesis, these data also suggest that a pharmacological intervention aimed at increasing plasmin in the CNS, or reducing the level of its inhibitor PAI‐1, could represent a new and alternative strategy to treat dementia associated with various neurological disorders. Nonetheless, before undertaking this type of study, the involvement of plasmin and its enzymes in dementia need to be investigated in larger cohorts of subjects affected by dementia of different origin.

## 5 LIMITATIONS

There are some limitations in the interpretation of our results. The number of subjects included is small due to the difficulty to recruit patients with FTLD and healthy subjects into the study. Moreover, we only included a single time point measurement at admission, thus we do not know how serum levels of PAI‐1 and tPA change during disease progression. Also, we did not measure tPA activity in serum and we do not know if the increase in PAI‐1 could have affected such activity while tPA serum levels were unchanged. For these reasons, our data must be considered as preliminary observations. More robust data with more complex study designs are needed before drawing definitive conclusions.

## 6 CONCLUSIONS

In summary, our data show that patients with dementia due to FLTD are characterized by increased PAI‐1 serum levels versus FLTD with MCI and controls. These data suggest that the plasmin system may be altered in patients manifesting dementia caused by different aetiology, with possible consequences on neuronal functions, including synaptic activity and cognition.

## AUTHOR CONTRIBUTIONS

**Francesco Angelucci:** Conceptualization (equal); data curation (equal); funding acquisition (equal); investigation (equal); methodology (equal); writing – original draft (equal); writing – review and editing (equal). **Katerina Veverova:** Conceptualization (equal); investigation (equal); methodology (equal); validation (equal); writing – original draft (equal); writing – review and editing (equal). **Alžbeta Katonová:** Conceptualization (equal); data curation (equal); formal analysis (equal); investigation (equal); methodology (equal); writing – original draft (equal); writing – review and editing (equal). **Martin Vyhnalek:** Data curation (equal); methodology (equal); supervision (equal); validation (equal); writing – original draft (equal); writing – review and editing (equal). **Jakub Hort:** Conceptualization (equal); funding acquisition (equal); project administration (equal); supervision (equal); validation (equal); writing – original draft (equal); writing – review and editing (equal).

## FUNDING INFORMATION

This study was supported by the Ministry of Health of the Czech Republic (grant no. NV19‐04‐00560) and by the project National Institute for Neurological Research (Programme EXCELES, ID Project No. LX22NPO5107) funded by the European Union (Next Generation EU). This study was also supported by EEA/ Norway Grants 2014–2021 and the Technology Agency of the Czech Republic (project number TO01000215).

## CONFLICT OF INTEREST STATEMENT

The authors declare that they have no conflict of interests.

## Data Availability

The data of this study are available from the corresponding author upon reasonable request.
